# The Therapeutic Potential of Anticoagulation in Organ Fibrosis

**DOI:** 10.3389/fmed.2022.866746

**Published:** 2022-05-16

**Authors:** Hanna Oh, Hye Eun Park, Min Su Song, HaYoung Kim, Jea-Hyun Baek

**Affiliations:** School of Life Sciences, Handong Global University, Pohang, South Korea

**Keywords:** fibrosis, coagulation, ECM, coagulation factors, hemostasis

## Abstract

Fibrosis, also known as organ scarring, describes a pathological stiffening of organs or tissues caused by increased synthesis of extracellular matrix (ECM) components. In the past decades, mounting evidence has accumulated showing that the coagulation cascade is directly associated with fibrotic development. Recent findings suggest that, under inflammatory conditions, various cell types (e.g., immune cells) participate in the coagulation process causing pathological outcomes, including fibrosis. These findings highlighted the potential of anticoagulation therapy as a strategy in organ fibrosis. Indeed, preclinical and clinical studies demonstrated that the inhibition of blood coagulation is a potential intervention for the treatment of fibrosis across all major organs (e.g., lung, liver, heart, and kidney). In this review, we aim to summarize our current knowledge on the impact of components of coagulation cascade on fibrosis of various organs and provide an update on the current development of anticoagulation therapy for fibrosis.

## Introduction

Fibrosis (organ scarring) denotes a pathological condition characterized by the elevation of interstitial fibrous tissue. The hallmarks of fibrosis include the presence and persistence of myofibroblasts and collagen deposits. Fibrosis is a consequence of failed tissue repair, often occurring due to a severe incurable, persisting, or repeated injury.

An injured tissue activates a tightly coordinated tissue repair program that is classically divided into four processes: (1) hemostasis, (2) inflammation, (3) proliferation, and (4) remodeling (1). Hemostasis induces vasodilation and platelet “plug” activation in response to an injury. Hemostasis activates blood coagulation, a complex cascade system that leads to fibrin clot formation in the damaged tissue. The fibrin clot minimizes bleeding as well as traps immune cells and pathogens facilitating immune response against infection (2). Hemostasis is accompanied by inflammation, a localized response to tissue damage. Hemostasis and inflammation affect each other: inflammation activates the hemostatic system that, in turn, controls inflammatory activity (3).

To date, studies have indicated that the persistence of fibrin in the matrix promotes fibrosis (4); increased levels of fibrinogen or impaired fibrinolysis increases collagen formation and subsequent fibrosis, while loss of fibrinogen or improved fibrinolysis ameliorates fibrosis (3–9). These findings led to the identification of coagulation factors as therapeutic targets in organ fibrosis. Indeed, growing evidence shows that the inhibition of blood coagulation is a promising intervention for the treatment of fibrosis across all major organs (5). However, the precise mechanistic link between fibrin and collagen formation still deserves further investigations.

## Mechanisms of Blood Coagulation

Blood coagulation is a cascade system of enzymes that collectively produce fibrin clot in damaged tissues. The fibrin clot provides a provisional matrix for tissue repair: the fibrin matrix acts as a reservoir of growth factors and proinflammatory cytokines, promoting leukocyte migration, and the accumulation, activation, and proliferation of fibroblasts (6).

**FIGURE 1:**
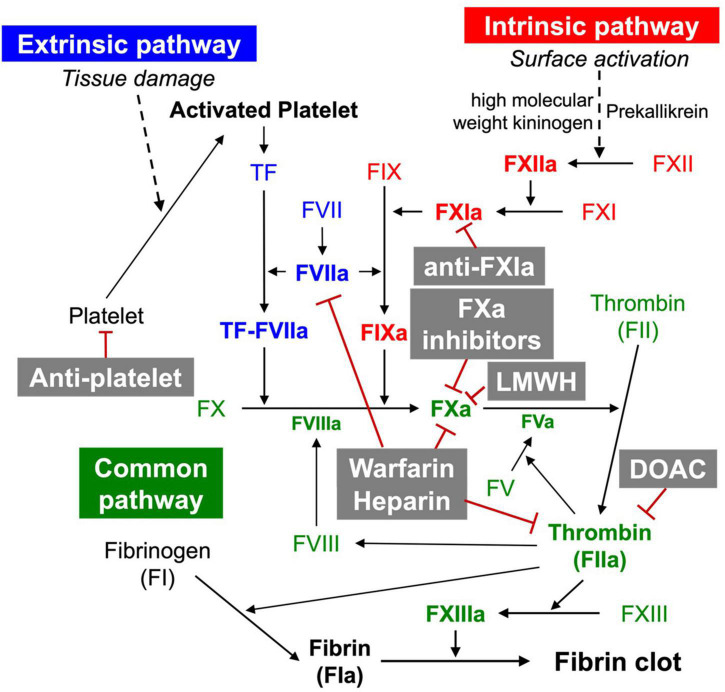
Coagulation cascade and anticoagulants.

The coagulation cascade is divided into three pathways: the intrinsic, extrinsic, and common pathways (Figure 1) (10). The extrinsic pathway consists of the extravascular tissue factor (TF) and coagulation factor VII/VIIa (FVII/FVIIa) (11). TF is a protein constitutively expressed in tissues (e.g., by smooth muscle cells, pericytes, and fibroblasts) (7). Once TF binds to circulating FVIIa, the TF:FVIIa complex is formed. The TF:FVIIa complex then hydrolyses FX to its active form FXa. Under inflammatory conditions, TF is additionally expressed by monocytes, neutrophils, endothelial cells, and platelets leading to pathologic outcomes (e.g., thrombosis) (12). The intrinsic pathway consists of FIX, FXI, and FXII. The intrinsic pathway is also called the contact activation pathway because coagulation factors are activated by an externally charged surface (e.g., collagen). The intrinsic pathway begins with the binding of three plasma proteins (FXII, prekallikrein, high molecular weight kininogen) to a surface (13). The subsequent activation of FXII to FXIIa converts FXI to FXIa. FXIa, in turn, activates FIX, which forms a complex with its cofactor FVIIIa, converting FX to FXa. In the common pathway, FXa and its cofactor FV activate prothrombin to produce thrombin (FIIa). Thrombin subsequently converts fibrinogen to form fibrin, and activates FXIII to FXIIIa, covalently crosslinking the fibrin strands to form a more stable fibrin network. Once thrombin is activated, the amplification of coagulation begins. This occurs through thrombin, which catalysis the activation of FV, FVII, FIX, FX, and FXI (14).

During normal tissue repair, fibrin is removed by a process, called fibrinolysis, mediated by the serine protease plasmin, which is converted from plasminogen by the urokinase-type (uPA) and tissue-type plasminogen activator (tPA) and regulated by plasminogen activator inhibitor-1 (PAI-1) (15). PAI-1 is induced inflammatory cytokines and growth factors (e.g., EGF, IL-1β, TGF-β) (8). Normally, PAI-1 is significantly elevated in fibrotic tissues, and PAI-1 deficiency protects organs from fibrosis in response to injury-related profibrotic signals (8).

Recent studies on the coagulation cascade have revealed that the function of the coagulation cascade is not limited to fibrin clot formation and prevention of blood loss, but it is also crucial in regulating the inflammatory response, tissue repair, and wound healing process (16).

## Coagulation Factors in Organ Fibrosis

### Coagulation Factors in Pulmonary Fibrosis

The tissue factor (TF)-FVII/VIIa complex has been studied as a potential mechanism by which the coagulation cascade contributes to pulmonary fibrosis development (17). TF expression is upregulated in the bronchoalveolar lavage fluid (BALF) of IPF patients and on type II pneumocytes of IPF patients (18, 19). Concerning IPF, the TF-FVII/VIIa complex has also been observed in the BALF obtained from patients with acute respiratory distress syndrome (ARDS); lung injury of which fluid leakage into the lungs may progress to a fibroproliferative response (20, 21). FVII has also been linked to intra-alveolar fibrosis, considered to be involved in the merging of alveolar walls observed in IPF, as it has been identified in intra-alveolar fibrosis patients (22, 23).

Increasing amounts of evidence supported that both FX/Xa and thrombin, other than their role in downstream coagulation cascade precursor activation, are involved in the fibro-proliferative response activation in IPF (23–27). Increased FXa expression has been reported in fibrotic lung tissue (28). The presence of FX activating procoagulant microparticles has been identified in the airways of IPF patients (29). Elevated thrombin levels in BALF samples obtained from pulmonary fibrosis patients have been reported as well (30, 31). It has been shown that FX/Xa and thrombin can cleave protease-activated receptors (PARs) for the activation of fibroblast proliferation and myofibroblast differentiation (32–35). FX/Xa and thrombin-mediated activation of PARs profibrotic effects also induce proinflammatory cytokine secretion and ECM deposition stimulation (26, 36).

The involvement of coagulation FVIII/VIIIc, FXIIa, and other prothrombotic factors may also play important roles in IPF. Elevated levels of FVIII/VIIIc were observed in IPF patients (35, 37). In one study, IPF patient lung fibroblasts were reported to have enhanced binding capacity for FXIIa, suggesting a potential role of FXIIa in fibrosis (38).

### Coagulation Factors in Hepatic Fibrosis

Several clinical studies have indicated that anticoagulation may be a promising therapeutic approach in hepatic fibrosis (39–43). Congruently, a meta-analysis of preclinical studies also provided strong evidence that anticoagulants alleviate hepatic fibrosis (44). Chronic liver diseases are often associated with portal vein thrombosis (PVT), and anticoagulation is a standard of care for treating PVT. Thus, studies have assessed the pharmacological safety of anticoagulants in patients with advanced chronic liver diseases (45–47).

Coagulation factors, FV, FVII, and FX are particularly prominent in hepatic fibrosis and cirrhosis (48–50). Due to the promotion of prothrombic state by these factors, fibrin(ogen) deposits may obstruct the hepatic sinusoids, which affect the blood flow and may set the base for fibrosis initiation or liver disease advancement (48). A connection between TF and hepatic fibrosis is suggested as fibrosis was reduced in transgenic mice lacking TF (51). In preclinical studies, FXa played an essential role in CCl4-induced hepatic fibrosis (52). Furthermore, FXa stimulated proinflammatory and profibrotic mediator production and thus activated hepatic stellate cells (HSCs) and the fibrogenic process (52). Also, TF signaling plays a direct role in CD68^+^ macrophage activation (53). Macrophages constitutively express and upregulate TF as macrophages mature (53). Kupffer cell polarization (M1) may be regulated and sustained by TF signaling (53).

### Coagulation Factors in Cardiac Fibrosis

A murine cardiac fibrosis model exhibited elevated levels of locally produced FXa compared to sham-operated mice. FXa is upregulated in the hypertrophic fibrotic cardiac interstitium (54). During cardiac fibrosis, PARs are activated by thrombin and FXa and induce cardiac fibroblast proliferation as well as myofibroblast activation (55). The infarction size of PAR-2 KO mice was significantly reduced after 30 min of ischemia on the heart, and the mRNA transcription of inflammatory genes, IL-1β, IL-6, and TNF-α, was attenuated in the heart of PAR-2 KO mice (56). However, transgenic mice that overexpressed PAR-1 on cardiomyocytes exhibited increased hypertrophy and cardiac remodeling, although whether PAR-1 directly affected myocardial infarction is unclear (57). Also, peptide agonists of PAR-1 and PAR-2 stimulated cardiac fibroblast proliferation. These collective data corroborated the role of PAR signaling mediated by coagulation factors, thrombin and FXa in cardiac inflammation, fibrotic response, and repair process.

### Coagulation Factors in Renal Fibrosis

The role of coagulation factors in kidney disease beyond normal hemostasis and thrombosis has been long suspected, and the increasing clinical utilization of anticoagulants targeting specific coagulation factors gain deeper insights into the mechanisms through which the coagulation system modulates renal health (58). Notably, increased TF expression has been detected in acute and chronic kidney diseases (59–64). Proinflammatory stimuli induce glomerular FV expression, whereas kidney IRI induces tubular fibrinogen expression (63, 65–67). Direct effects of coagulation proteases such as thrombin or FXa on glomerular cells have long been known, implying receptor-dependent modulation of intracellular signaling pathways (63, 68). The expression of PARs in renal cells provides a molecular link between coagulation factors and renal cell function (69–72).

## Anticoagulants in Organ Fibrosis

### Heparin and Warfarin

Heparin and its low-molecular-weight derivatives (LMWHs, e.g., enoxaparin, nadroparin tinzaparin) are anticoagulants widely used in the prevention of blood clots (e.g., during hemodialysis, surgery) and in the prophylaxis and treatment of venous thromboembolism and myocardial infarction. Heparin inhibits blood coagulation by increasing the activity of antithrombin III, an enzymatic inhibitor of thrombin, FXa, and other proteases (Figure 1). Although heparin does not directly dissolve already formed clots, heparin may support the body’s natural clot lysis mechanisms. Therefore, several studies have assessed the anti-fibrotic activity of heparin and LMWHs using preclinical models. Consequently, researchers have found that heparin and LMWHs alleviate experimental pulmonary (73, 74) and hepatic (75–79), as well as endomysial fibrosis (80). In line with this, clinical studies corroborated the therapeutic potential of heparin and LMWHs in organ fibrosis by demonstrating that heparin and LMWHs inhibit collagen proliferation in the liver with chronic hepatitis B virus infection (39). Despite positive perspectives, clinical trials of heparin and LMWHs for organ fibrosis have been so far without success. Anti-fibrotic therapies with heparin and LMWHs appeared to pose more risks than benefits (81).

Warfarin is another widely used anticoagulant. As a vitamin K antagonist, warfarin inhibits vitamin K epoxide reductase, which is essential for the reactivation of vitamin K_1_ and, in turn, for ensuring the activity of vitamin K_1_-dependent coagulation factors such as thrombin, FVII, FIX, and FXa (82) (Figure 1). In animal studies, warfarin showed protective effects on hepatic fibrosis following congestive hepatopathy induced by partial ligation of the inferior vena cava (82) as well as following a CCl_4_ challenge (83). Clinical studies revealed that warfarin and LMWHs ameliorate portal vein thrombosis in patients with liver cirrhosis reducing *de novo* occurrence, improving new hepatic decompensation, and survival of patients with advanced disease (42). Other studies show that warfarin, heparin, and LMWHs reduce the proliferation and activation of HSCs limiting fibrotic areas (75). However, it remains to be clarified whether warfarin, heparin, and LMWHs have direct effects on HSCs, or the suppression of HSCs is rather a consequence of the anti-fibrotic activities of the anticoagulants. Warfarin does not appear to be effective in IPF. Warfarin was not beneficial in experimental model of IPF (24). Congruently, a clinical study of warfarin in IPF was suspended due to a low probability of benefit and an increase in mortality (84). However, this was contrary to the result of a precedent clinical study, where patients administered with warfarin in combination with prednisolone demonstrated significantly improved survival rates in acute IPF exacerbations as compared to the group treated with prednisolone alone (85).

### Anti-platelet

Anti-platelet agents, e.g., aspirin, clopidogrel, ticagrelor, and prasugrel, serve as potential candidates for thrombosis treatment. Activated platelets, in combination with FVIIa, mark the initiation of the coagulation cascade (Figure 1). It is unclear whether TF is expressed by platelets (86). However, the formation of the TF-FVIIa complex may occur on platelets, potentially after absorbing microvesicles containing TF (86–88). Platelets are known for their role in accelerating the activation of coagulation cascade by setting the base for the prothrombinase complex to form (89). Platelets, by aggregating to the site of fibrosis, may worsen organ destruction (90). Research has shown that anti-platelet agents are also effective in attenuating organ fibrosis.

Aspirin inhibits platelets by acetylating serine_529_ in the active site of the platelet cyclooxygenase (COX) (91). By changing the structure of the active site, the platelet-dependent thromboxane formation is inhibited (91). Aspirin can reduce the severity of hepatic fibrosis and renal impairment (75, 92). Significant improvement in hepatic fibrosis was observed at both low- and high-dose levels of aspirin-treated groups, with a remarkable improvement in the high-dose aspirin-treated group (75). In the case of CKD, aspirin has beneficial effects in mild renal impairment conditions compared to moderate renal impairment conditions (92).

Clopidogrel is another anti-platelet drug used to treat thrombosis and has found applications in the treatment of certain types of fibrosis in mouse models (93). Clopidogrel works as an anti-platelet drug by preventing ADP from stimulating P2Y_12_, the purinergic receptor of platelets (94). Clopidogrel combats fibrosis through mechanisms related to angiotensin II, which has had reports of being a pro-fibrotic factor (93). Therapeutic effects of clopidogrel when combating fibrosis are assumed to occur by inhibiting angiotensin II-induced accumulation of myofibroblasts (93). As a treatment of hepatic fibrosis, clopidogrel shows a more promising effect compared to dabigatran, a direct oral anticoagulant, indicated by the difference between the hydroxyproline expressions: the main component of collagen and a potential fibrosis marker (95).

Other platelet inhibitors are currently under development for the treatment of fibrosis. Ticagrelor and prasugrel are P2Y_12_ antagonists and may also work as platelet antagonists. These drugs bind to P2Y_12_, the adenosine diphosphate (ADP) receptor of platelets, and prevent blood coagulation (93). A recent study has shown that ticagrelor may have a positive effect in ameliorating pulmonary fibrosis in rats (96). Likewise, prasugrel may have positive effects on relieving hepatic fibrosis (90). However, further research is necessary for these drugs to be used in human clinical trials as it is unclear, whether the effects in animal studies would be translatable to patients is unclear (96).

### Direct Oral Anticoagulants

Direct oral anticoagulants (DOAC)—dabigatran, rivaroxaban, apixaban, edoxaban, and betrixaban are anticoagulation pharmacotherapies used for the prevention of thrombosis (Figure 1). DOACs are categorized into two main classes: direct FXa inhibitors (rivaroxaban, apixaban, edoxaban, and betrixaban) and direct thrombin inhibitors (dabigatran).

Rivaroxaban, apixaban, and edoxaban are direct FXa inhibitors that inhibit FXa by directly binding to the active site of FXa. Fondaparinux is an indirect FXa inhibitor that binds to antithrombin and changes its conformation which leads to inhibition of FXa (97). Dabigatran is a direct thrombin inhibitor that binds directly to active sites of thrombin and inhibits its activity (98). Recent studies revealed a correlation between thrombin and FXa with organ fibrosis, and the effectiveness of DOAC in treating organ fibrosis has emerged as a novel treatment of organ fibrosis. In an atrial fibrosis-induced model by transverse aortic constriction, the expression of TNF-α, IL-1β, and IL-6 was suppressed by treating rivaroxaban (99). In addition, collagen III, connective tissue growth factors, and TGF-β, which are upregulated during fibrosis development, were decreased by rivaroxaban treatment. Rivaroxaban treatment also inhibited FXa-mediated phosphorylation of NF-κB p65 and STAT3, which are important mediators of myofibroblast differentiation and fibrosis development (54). In the myocardial ischemia (MI)-induced fibrosis model, expression of collagen 1a1 and collagen 3a1 was significantly decreased after a high dosage of direct FXa inhibitor apixaban which indicates the efficacy of apixaban in attenuating MI-induced fibrosis (100). In unilateral ureteral obstruction (UUO) mice, which is the renal tubulointerstitial model, edoxaban treatment attenuated renal interstitial macrophage infiltration and release of inflammatory cytokines. Also, the expression of collagen II and III, TGF-β, and α-smooth muscle actin (SMA) was significantly attenuated with edoxaban treatment (101). Treatment of fondaparinux to kidney fibrosis, which was induced in mice by IRI, reduced macrophage infiltration. Also, proinflammatory IL-1β mRNA levels decreased in fondaparinux-treated models (67).

The efficacy of direct thrombin inhibitors in treating organ fibrosis has been revealed by dabigatran treatment. Both early and late treatment of dabigatran attenuated the development of bleomycin-induced pulmonary fibrosis by significantly reducing thrombin activity and inflammatory cells and protein concentrations in the bleomycin-induced pulmonary fibrosis model (102). Additionally, dabigatran inhibited thrombin-induced cell proliferation, α-SMA expression and organization, and the *in vitro* production of collagen and connective tissue growth factor (CTGF) by lung fibroblasts (103). Dabigatran attenuates cardiac fibrosis by improving coronary flow reserve and global cardiac function potentially by inhibiting thrombin activity and down-regulating PAR-1 expression (104). In the UUO model, dabigatran significantly inhibited UUO-induced type 1 collagen and tubulointerstitial fibrosis by inhibiting thrombin-activated PAR-1 expression in fibrotic kidneys (105). In hepatic IRI mice, dabigatran treatment significantly improved liver histological damage, induced sinusoidal protection, and provided both antiapoptotic and anti-inflammatory effects (106).

Rivaroxaban, co-administered with aspirin alone or with aspirin plus clopidogrel or ticlopidine, is indicated in the prevention of atherothrombotic events in patients with acute coronary syndrome (107). In clinical studies, drugs that pharmacologically target FX were effective in patients with cirrhosis of the liver. Rivaroxaban is currently in Phase III trials for treating patients with liver cirrhosis.

## Discussion

A large body of evidence has accumulated over the past decades to support the potential of anticoagulants as a promising therapeutic strategy for organ fibrosis. The literal meaning of the term “fibrosis” is “fibrous growth or development in an organ”; that of “fibrin” is “fibrous substance.” Although these terms appear to be semantically related, not much attention was previously paid to the biological link between fibrosis and coagulation. Preclinical and clinical studies have consistently demonstrated that the inhibition of coagulation may be beneficial in organ fibrosis. However, there have been so far only a few anticoagulation drug approvals/trials for the treatment of organ fibrosis *per se*, although anticoagulants are currently being used for complications, which are frequent in organ fibrosis. Anticoagulants are effective but have serious adverse effects making us extra cautious when expanding the application areas of these therapies. To date, our mechanistic understanding of how coagulation contributes to organ fibrosis is still largely unclear. Deeper insights into the precise mechanistic and functional role of coagulation in fibrotic development will allow us to better weigh the benefits and risks of anticoagulants in anti-fibrotic therapy.

## Author Contributions

All authors listed have made a substantial, direct, and intellectual contribution to the work, and approved it for publication.

## Conflict of Interest

The authors declare that the research was conducted in the absence of any commercial or financial relationships that could be construed as a potential conflict of interest.

## Publisher’s Note

All claims expressed in this article are solely those of the authors and do not necessarily represent those of their affiliated organizations, or those of the publisher, the editors and the reviewers. Any product that may be evaluated in this article, or claim that may be made by its manufacturer, is not guaranteed or endorsed by the publisher.

## Abbreviations

ARDS: acute respiratory distress syndrome
BALF: bronchoalveolar lavage fluid
ECM: extracellular matrix
IPF: idiopathic pulmonary fibrosis
IRI: ischemia-reperfusion injury
LMWH: low-molecular-weight heparin
PAI: plasminogen activator inhibitor
PAR: protease-activated receptor
SMA: smooth muscle actin
TF: tissue factor
MMP: matrix metalloprotease
TIMP: tissue inhibitor of metalloprotease.
